# Editorial: Energy-producing organelles and the nucleus: a phenomenal genomic friendship

**DOI:** 10.3389/fgene.2023.1230032

**Published:** 2023-06-23

**Authors:** Dmytro V. Gospodaryov, J. William O. Ballard, M. Florencia Camus, Rob DeSalle, Michael R. Garvin, Uwe Richter

**Affiliations:** ^1^ Department of Biochemistry and Biotechnology, Vasyl Stefanyk Precarpathian National University, Ivano-Frankivsk, Ukraine; ^2^ School of Biosciences, University of Melbourne, Parkville, VIC, Australia; ^3^ Department of Environment and Genetics, School of Agriculture, Biomedicine and Environment, La Trobe University, Melbourne, VIC, Australia; ^4^ Department of Genetics, Evolution and Environment, University College London, London, United Kingdom; ^5^ Institute for Comparative Genomics, American Museum of Natural History, New York, NY, United States; ^6^ Division of Invertebrate Zoology, American Museum of Natural History, New York, NY, United States; ^7^ Computational Predictive Biology, Oak Ridge National Laboratory, Oak Ridge, TN, United States; ^8^ Wellcome Centre for Mitochondrial Research, Faculty of Medical Sciences, Newcastle University, Newcastle Upon Tyne, United Kingdom; ^9^ Organismal and Evolutionary Biology Research Programme, Faculty of Biological and Environmental Sciences, University of Helsinki, Helsinki, Finland

**Keywords:** mitochondria, chloroplast, endosymbioses, mitonuclear coevolution, mitonuclear coadaptation, *Drosophila melanogaster*, *Caenorhabditis elegans*, mitochondrial genome

Mitochondria and chloroplasts are energy-producing organelles that are considered to be descendants of bacteria that were either phagocytosed by or had invaded proto-eukaryotic cells. Mitochondria are essential for obligatory aerobic respiration in animal and plant cells, whereas plants are incapable of synthesizing organic compounds without chloroplasts. Notably, mitochondria and chloroplasts retained some of the gene expression machinery of their bacterial ancestors and this machinery included a part of the original genome, bacterial-like ribosomes, as well as parts of the replication, transcription, and translation factors, similar to those that operate in contemporary bacteria. However, mitochondrial transcription and replication machineries are largely of bacteriophage origin and were acquired from an ancestor of a T3/T7-like bacteriophage that was likely present in the ancestral α-proteobacterium at the time of the mitochondrial endosymbiosis (Filée and Forterre, 2005; Shutt and Gray, 2006). Plant mitochondrial genomes are more complex, having more genes, than either fungal and streamlined animal mitochondrial DNA genomes. The endosymbioses between a eukaryotic ancestor and ancestors of mitochondria and chloroplasts are mutually beneficial. Nevertheless, each participant lost independence in order to acquire other benefits that allowed them to propagate and occupy new niches. The eukaryotic ancestor contributed substantial and beneficial metabolic pathways to the symbiont. However, it became vulnerable to damages done to the symbiont. In turn, the symbionts allowed the host cell to encode essential parts of their proteome. Essentially, the symbionts forced the host to include them into the host’s evolutionary trajectories. For example, complexes I, III, IV, and V of the mitochondrial oxidative phosphorylation (OXPHOS) system are composed of subunits encoded by both nuclear and mitochondrial genomes. The assembly of the complexes occurs with the assistance of nuclear-encoded factors. Mutations that affect coordination between the nuclear-encoded and mitochondria-encoded subunits of the complexes could profoundly change OXPHOS effectiveness (Couvillion et al., 2016). Nuclear-encoded proteins are essential for the transcription of mitochondrial DNA and translation of mitochondrial messenger RNA. Therefore, a discordance in operation of the genomes decreases fitness of the whole organism.

Mitochondria generate a number of signaling molecules such as reactive oxygen species (ROS), acetyl coenzyme A, dicarboxylic acids (α-ketoglutarate, succinate, and fumarate), and regulate turnover of others such as calcium, adenylates, and guanylates. This means that mitochondria are involved in a complex organismal response to the changes in its environment. The changes are sensed by cellular signaling machinery and the signal is transduced to the nuclear-encoded transcription factors that, in turn, shape the mitochondrial proteome. This algorithm is confirmed by Rodríguez et al., who showed that larvae of the fruit fly, *Drosophila melanogaster*, strains with specific mitochondrial haplotypes respond differently to diet with N-acetyl cysteine.

Numerous theories postulate a key role for energy-producing organelles, particularly mitochondria, in the origin of multicellularity and sexual recombination (Garg and Martin, 2016; Radzvilavicius et al., 2016). Indeed, mitochondria provided ATP for a multitude of functions that are not present in prokaryotes, such as complex intercellular communication, neural impulses, exchange of metabolites or division of labor. In the majority of organisms, the egg has an abundance of mitochondria whereas a sperm has very few. Interestingly, intracellular mitochondria-like insect parasites such as *Wolbachia* maximize their survival, affecting males’ fertility and changing the ratio between females and males in the host’s population (Werren et al., 2008). It allows parasites to survive in the germline of females. The opposite situation was shown in the case of mismatch between nuclear and mitochondrial genomes that are believed to coevolve. Natural populations of the nematode *Caenorhabditis elegans* contain a very small proportion of males. However, mutations in mitochondrial genes that encode subunits of the respiratory chain strongly affect sex determination in *C. elegans* (Bever et al.).

It is remarkable that nuclear genes are inherited from both parents and undergo recombination further, whereas mitochondrial genes have predominantly maternal inheritance (Torres-Gonzalez and Makova). Surprisingly, such peculiarities in inheritance of nuclear and mitochondrial genomes enable a situation where an individual obtains a mitochondrially-encoded proteome that is not well coordinated with its nuclear-encoded proteome (Torres-Gonzalez and Makova). Basically, it may result in the above-described situations when mitochondrial genes influence a response to environmental changes or affect the natural ratio between sexes.

A frequent cause of the above-mentioned mitonuclear mismatches is that a subset of the subunits of the mitochondrial OXPHOS system complexes are encoded by nuclear DNA whereas others are encoded by mitochondrial DNA. We expect formation of the perfect match and coordination between nuclear- and mitochondrial-encoded subunits due to long-term coevolution.

Nuclear-encoded replication machinery genes, namely, DNA polymerase γ, helicase, single strand binding proteins (SSB), and other factors should also coevolve with mitochondrial-encoded proteins. Ciesielski et al. show that DNA polymerase γ can directly interact with mitochondrial SSB that, in turn, decreases binding of DNA polymerase γ to single-stranded DNA without primers attached. The same research team discovered that the iron-sulfur (Fe-S) cluster in the N-terminal domain of the fruit fly’s mitochondrial DNA helicase is bound to the membrane. They have also found that this Fe-S cluster is delivered by the iron-sulfur cluster assembly factor IND1 (iron-sulfur cluster assembly protein known to be required specifically for NADH dehydrogenase), which was previously shown to take part in the assembly of the complex I (So et al.). This adds complexity to our understanding of integration of the host (a proto-eukaryotic cell) and its symbiont (mitochondria) proteomes.

The articles in the Research Topic “*Energy-Producing Organelles and the Nucleus: A Phenomenal Genomic Friendship*” represent a number of excellent examples that show consequences of mitonuclear discordance. The article of Triant and Pearson explores the methods to study mitonuclear interactions. Finally, the article of Christian et al. reviews mechanisms of protein transport into non-green plastids, a good model to study how the host proteins are delivered to the organelle of endosymbiotic origin. It is tempting to see the continuation of the studies conducted by the contributors and further development of stories started on the pages of *Frontiers in Genetics*. Indeed, we further learn that the BAR and COX lines with mitonuclear mismatch respond to the N-acetyl cysteine treatment by suppression of complex I-linked respiration (Camus et al., 2023). We also see a deeper study of protein-protein interactions in mitochondrial DNA replication machinery in insect cells (Plaza et al., 2023).

The general theme of the interaction of organellar and nuclear genomes is one of cooperation. This cooperation necessitates compensatory evolution between two genomes with different mutational processes and vastly different modes of inheritance making for very strange bedfellows indeed.

In general, *Frontiers in Genetics* issues up to hundred Research Topics that explore different aspects of the endosymbiotic genome architecture and intergenomic communication. Among them, and in addition to our Topic we would recommend readers to find even more in the parallel collections of articles such as “Impact of Cytonuclear Incompatibilities on the Evolution of Species,” “Mitochondrial Genomes and Mitochondrion Related Gene Insights to Fungal Evolution,” “Evolution of Plant Mitochondrial Genomes,” “Evolution of Mitochondrial Genomes,” “Mitochondrial Genetics and Epigenetics,” “Interorganelle communication in aging,” and others.
